# Identification and Management of Hyperprolactinaemia in Patients With Intellectual Disabilities Who Are Prescribed Antipsychotic Medication: A Retrospective Audit

**DOI:** 10.1192/bjo.2024.574

**Published:** 2024-08-01

**Authors:** Soracha Healy, Rupal Patel

**Affiliations:** 1 South West London and St George's NHS Mental Health Trust, London, United Kingdom; 2 Your Healthcare, London, United Kingdom

## Abstract

**Aims:**

Hyperprolactinaemia has long-term complications including reduced bone mineral density (BMD). People with an intellectual disability (ID) have a greater burden of disease and reduced life expectancy compared with the general population, including an increased risk of osteoporosis and fractures. There is a higher prevalence of antipsychotic prescriptions in people with ID which increases the risk of hyperprolactinaemia. Therefore, regular serum prolactin monitoring is important in this group. The aims of this audit were:

1. To identify how many patients with ID are prescribed antipsychotic medication and of these, how many have had prolactin levels measured in the last 12 months.
2. To identify how many patients had elevated prolactin levels (>530 mIU/L).
3. To identify if results had been managed as per current guidelines.

**Methods:**

Data was reviewed from the Richmond and Kingston psychiatry caseloads using the electronic patient record, Care Notes. Each patient was reviewed against the inclusion criteria of diagnosis of ID and currently prescribed antipsychotic medication.

125 patient records were reviewed on Care Notes. 50 patients were excluded as they were not prescribed an antipsychotic medication. The remaining 75 patients met the inclusion criteria.

**Results:**

75 patients were prescribed an antipsychotic. Of the 10 different antipsychotics prescribed, the most common were risperidone (50.7%) and olanzapine (30.7%). Of those prescribed an antipsychotic, 39 (52.0%) had their prolactin levels measured in the last 12 months.

The prolactin levels measured ranged from 82 mIU/L to 4890 mIU/L. 16 (41.0%) patients had elevated prolactin levels. In those with elevated prolactin, 68.8% were prescribed risperidone.

Of the patients with elevated levels, 81.3% had their results discussed with them and treatment options considered. The majority of patients were monitored and screened for symptoms. In some cases, psychotropic medication was reduced with a view to stopping and others continued to be monitored. Two patients were prescribed aripiprazole 2.5mg as an adjunct.

Those who had not had their prolactin levels discussed were all awaiting appointments as the blood tests had been taken recently.

**Conclusion:**

A key area identified is how to increase uptake of blood tests in this patient group. Closer liaison with GP surgeries and proactive discussions with patients about the importance of screening for hyperprolactinaemia may help to improve outcomes. Referral to our in-house needle desensitisation service may also be helpful.

There is also scope for future research regarding the management of hyperprolactinaemia in the ID population due to the increased risk of reduced BMD.

